# Comparison of Outcomes in Superior Canal Dehiscence Surgery Using Either Transmastoid or Middle Fossa Surgical Approaches: A Systematic Review and Meta-Analysis

**DOI:** 10.1177/19160216261435611

**Published:** 2026-03-30

**Authors:** Matthew Urichuk, Jason Azzi, Ben Woldu, Armon Hadian, Jordan Hochman

**Affiliations:** 1 Max Rady College of Medicine, University of Manitoba, Winnipeg, MB, Canada; 2 Department of Otolaryngology – Head and Neck Surgery, University of Manitoba, Winnipeg, MB, Canada

**Keywords:** otology, superior semicircular canal dehiscence, transmastoid, middle fossa, outcomes

## Abstract

**Importance:**

Superior semicircular canal dehiscence syndrome (SSCDS) can manifest with significant vestibular and auditory symptoms. For patients with debilitating SSCDS symptoms, surgical repair can be performed using a middle cranial fossa (MCF) or transmastoid (TM) approach, however, comparisons of outcomes between surgical approaches are unclear.

**Objective:**

To assess outcomes in SSCD repaired using either MCF or TM surgical approaches.

**Design:**

Systematic review and meta-analysis.

**Setting:**

A systematic search of all articles reporting on the presence of outcomes of SSCD repair in Medline, Embase, CINAHL, Scopus, and Web of Science databases.

**Participants:**

1130 patients with SSCDS reported in 34 manuscripts.

**Exposure/Intervention:**

Surgical repair of SSCD via MCF or TM approaches.

**Main Outcome Measures:**

Rates of post-operative improvement in subjective auditory and vestibular symptoms as well as changes in objective audiometric measures [air-conduction (AC), bone-conduction (BC), and air-bone gaps (ABG) pure-tone averages].

**Results:**

Patients were highly likely (60%-95%) to report improvement in audiological and vestibular symptom improvement following either technique. No differences were observed between surgical approaches, with the exception of oscillopsia [91.1% vs 61.7%; aOR = 6.40; 95% CI = (1.66-24.66); *P* = .007]. No significant post-operative objective changes in audition were observed (AC thresholds, BC thresholds or ABG).

**Conclusion:**

Outcomes of SSCD surgery are excellent. Most patients report significant improvement or resolution of their pre-operative symptoms, with only minor differences in symptom resolution observed across approaches.

**Relevance:**

Our results provide evidence that surgical correction of SSCD through MCF and TM approaches is effective. supporting individualized decision-making based on surgeon experience and patient factors.

## Graphical abstract

**Graphical Abstract:**
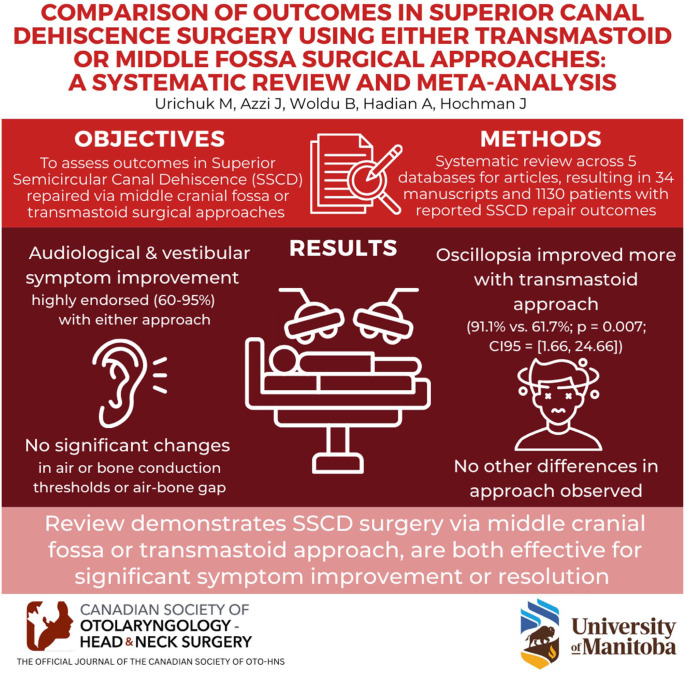

## Key Messages

- Surgical repair of superior semicircular canal dehiscence syndrome (SSCDS) using either the transmastoid (TM) or middle cranial fossa (MCF) approach results in high rates of vestibular and auditory symptom improvement.
- Post-operative auditory and vestibular symptom improvement was comparable between approaches, with only minimal differences observed.
- Average post-operative audiometric PTA thresholds were similar to pre-operative levels.
- Choice of surgical approach should be decided based on the surgeon’s preference and the individual patient factors.

## Introduction

Superior semicircular canal dehiscence (SSCD) refers to a dehiscence of the bony capsule that overlies the superior semicircular canal.^1^ A subset of individuals with a SSCD experience a variety of auditory and vestibular symptoms, which are hypothesized to be attributable to a third mobile window altering the biomechanics of the inner ear, known as SSCD syndrome (SSCDS).^2,3^

SSCDS symptoms typically include pressure- or sound-induced vertigo, oscillopsia, pulsatile tinnitus, and bone-conduction (BC) hyperacusis/autophony.^2^ Patients may also report other vertiginous and non-vertiginous dizziness symptoms such as aural fullness, non-pulsatile tinnitus, and subjective hearing loss.^3-5^ Along with characteristic symptoms, diagnosis of SSCDS requires confirmation of the dehiscence on temporal bone computed tomography (CT) and generally other diagnostic findings, including suprathreshold BC on audiometry, abnormal vestibular evoked myogenic potentials (VEMP), or nystagmus evoked by either sound, middle ear pressure changes, or intracranial pressures.^2^

For patients with debilitating SSCDS symptoms, the dehiscence can be surgically plugged, resurfaced, or capped.^6^ Typically, the dehiscence is surgically accessed using a middle cranial fossa (MCF) approach or a transmastoid (TM) approach. The traditional MCF approach requires a craniotomy and involves manipulation and retraction of the temporal lobe, providing direct visualization of the dehiscence.^5,7^ Further, owing to the approach, it requires a short hospitalization for close observation. The TM approach does not require a large craniotomy or the same degree of manipulation of the temporal lobe. It is often a day procedure. However, the approach does limit visualization and access to the dehiscence.^8^ Given the varying benefits and drawbacks of each surgical approach. It is imperative to determine if there is a difference in outcome based on the approach to inform best surgical practice.

Although post-operative outcomes in SSCDS surgery using both approaches have been reported, there is no recent systematic assessment of post-operative symptom improvement rates and audiometric outcomes. The objective of this systematic review and meta-analysis is to assess outcomes in patients with SSCDS who underwent surgical repair using either MCF or TM surgical approaches.

## Materials and Methods

A systematic literature review was conducted to identify studies that assessed pre- and post-operative outcomes in individuals with SSCDS. The search included published articles in Medline, Embase, CINAHL, Scopus, and Web of Science databases. The search included all articles from the inception of each database until March 2025. Search terms centered around superior canal dehiscence, the 2 surgical approaches being assessed (TM and MCF approach), and symptoms that are commonly reported to be associated with SCDS. The full search string can be found within Supplemental Material 1. This review was prospectively registered on PROSPERO (CRD42023432479). For inclusion, studies were required to report: (1) the presence of signs and/or symptoms pre-operatively and (2) whether there was an improvement and/or resolution of signs or symptoms post-operatively. Conference abstracts, review articles, non-human, non-English and duplicate studies were removed. Studies were excluded if fewer than 10 ears were reported on or outcomes could not be split by surgical approach. If multiple studies were completed on the same cohort of patients as identified by the manuscripts’ senior author, only the largest study was included.

**Figure 1.**
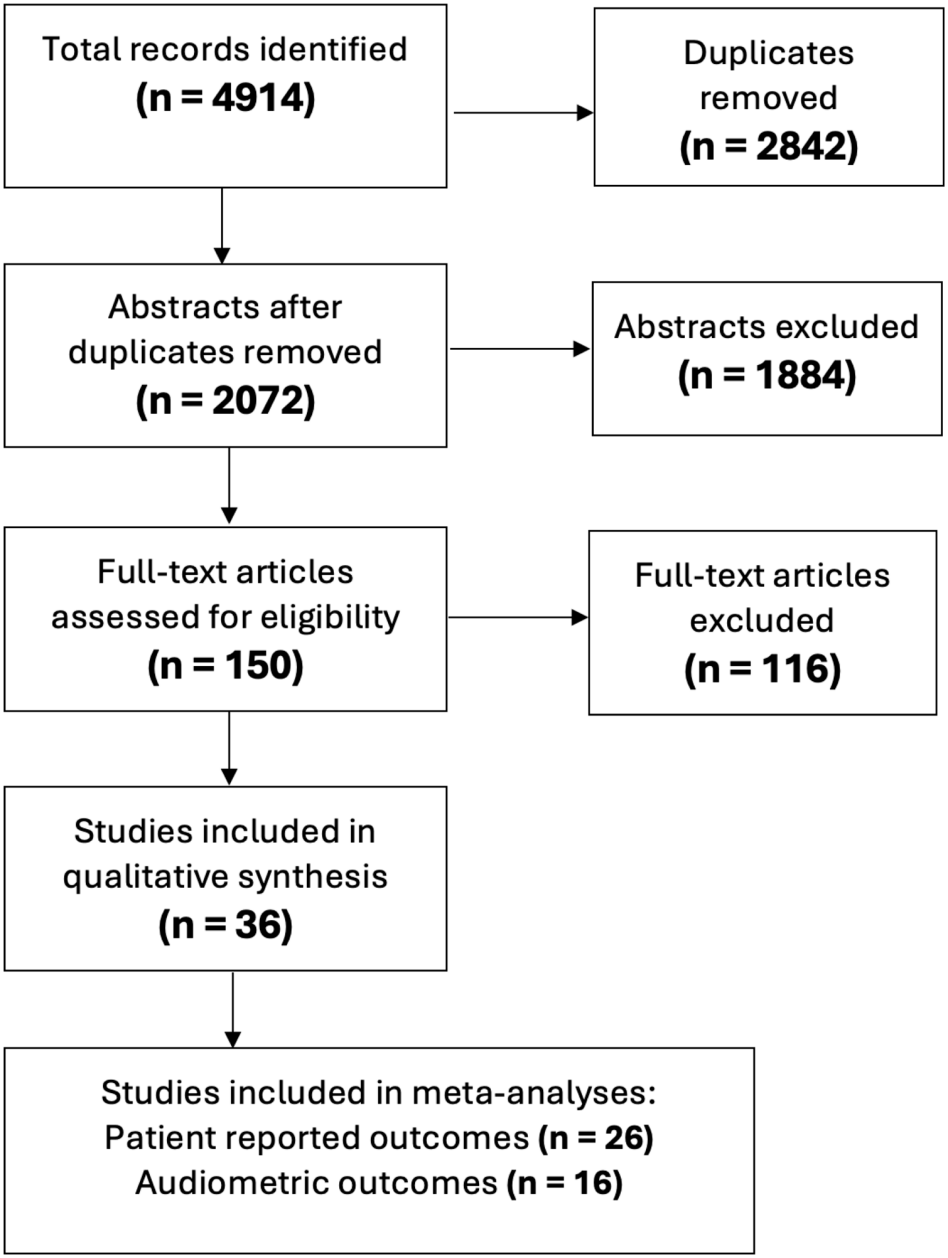
Flow diagram of study selection and eligibility.

Abstract screening was completed by 2 independent reviewers based on the relevance of the title and abstract (Figure 1). Studies were found to be eligible after full-text assessment. Any discrepancies between reviewers were resolved through consensus discussion with a third author. The systematic review followed the Preferred Reporting Items for Systematic Reviews and Meta-Analyses guidelines.^9^

Demographic information, surgical approach, the number of patients reporting specific pre-operative symptoms and the number with post-operative symptom improvement/resolution were recorded. Specific symptoms recorded were: subjective hearing loss, aural fullness, autophony, tinnitus, vertigo, oscillopsia, Tullio phenomenon, Hennebert sign, and non-vertiginous dizziness (ie, dizziness, imbalance, and unsteadiness). If reported within a study, Dizziness Handicap Inventory (DHI), cervical VEMP (cVEMP) outcomes, and audiometric air-conduction (AC), BC, and air-bone gap (ABG) pure-tone averages were collected. Only studies that reported Pure-tone averages (PTAs) or audiometric data in which the PTA could be calculated were included. If audiometric data was only available within a graph, the manuscript was excluded due to the inability to determine thresholds. Bias and overall quality of evidence were assessed for each included study using the Newcastle-Ottawa Scale.^10^

Meta-analyses were conducted on rates of post-operative symptom improvement for each recorded symptom and for audiometric outcomes. Symptom improvement was analyzed using a generalized logistic mixed model in R using the “metafor” package.^11^ These models included a binomial fixed effect of surgical approach (TM/MCF) and a random intercept for included studies. Symptomatic improvement was coded as a binomial variable (not improved vs improved/resolved). Pooled estimates of the proportion of patients experiencing post-operative symptom improvement/resolution are reported. Specific audiometric outcomes assessed were AC PTA, BC PTA, and ABG PTA. Mixed linear models assessed the mean difference between post- and pre-operative PTAs with a fixed effect for surgical approach (TM/MCF) and a random intercept for studies. Pooled estimates of the change in mean audiometric results (dB) are reported. For all outcomes within the meta-analyses, heterogeneity between studies was assessed using Cochrane’s *Q*-statistic.

## Results

A total of 2072 unique studies were identified and reduced to 150 studies after initial title and abstract screening. Thirty-four manuscripts reported pre- and post-operative outcomes met all inclusion criteria. Assessment of quality was completed on each study (Supplemental Material 2).

These studies included a total of 1130 patients, 741 (65.6%) who underwent SSCD repair using an MCF approach and 389 (34.4%) who underwent repair using a TM approach (Table 1).^12-43^ Follow-up duration varied significantly across studies, ranging from 1 month post-operatively to 4 years following the surgery. Over half of the patients undergoing SSCD repair via the MCF approach (55.3%; n = 410/741) came from a single study, conducted by Yang et al.^34^

**Table 1.** Baseline Characteristics for All Included Studies. Total Number of Patients Reported Is the Number With Subjective Outcomes Reported.

Study	N patients	N ears	Mean age (range), y	Male:female	Mean post-operative follow-up (mo)
Trans-mastoid					
AlAfif (2019)	10	12	52 (NR)	1:9	48 (NR)
Allsopp (2020)^a^	10	10	53.8 (23-76)	2:8	3 (NR)
Banakis-Hartl (2018)^a^	17	19	53 (38-77)	6:11	9 (2-24)
Beyea (2012)	16	16	NR	NR	NR
Bogle (2013)	20	20	48^b^ (36-80)	6:14	4 (4-21)
deWolf (2021)	52	55	47 (29-63)	24:31	11.2 (2-117)
Eberhard (2024)	13	16	45^b^ (34-62)	6:8	3 (NR)
Ellsperman (2021^a^, 2022)	25	28	55 (NR)	10:18	2.47 (0.27-8.67)
Gersdorff (2022)	27	30	52 (27-74)	11:16	21.4 (3-108)
Kontorinis (2021)	11	12	41.17 (32-65)	5:6	3 (10-14 wk)
Lundy (2011)	37	37	55.8 (35-98)	11:26	NR (3-NR)
Ma (2015)	10	10	42.1 (27-55)	7:3	33.8 (9-56)
Mehta (2022)^a^	36	36	53.1 (24-79)	18:18	27.2 (8-74)
Nieto (2021)	9	9	55 (33-68)	6:3	NR (6-156)^c^
Nogueira (2018)	21	22	NR	NR	38.4 (NR)
Remenschneider (2015)^a^	23	23	50 (18-76)	23:28	12 (3-39)
Rodgers (2016)^a^	15	15	48 (37-61)	11:18	NR
Schwartz (2019)	47	53	48 (NR)	22:25	12.1 (NR)
Shaul (2024)	23	24	54 (23-74)	8:15	12 (NR)
Tooker (2024)	38	38	50.5 (43-66)	15:25	9.5 (NR)
Van Haesendonck (2016)	12	12	54.1	6:6	1 (1-12)
Wolfovitz (2019)	8	8	49.8 (21-66)^c^	NR	14.1 (7-46)^c^
Zhao (2012)	10	11	46 (31-76)	4:6	NR (3-12)
Middle cranial fossa					
Baxter (2019)	34	34	41.1 (NR)	12:22	NR
Ellsperman (2021^a^, 2022)	21	24	43 (NR)	8:13	36.4 (3.5-154)
Goddard (2014)	23	24	52.2 (NR)	9:14	19.9 (NR)
Lee (2020)	11	11	49.2 (26-61)	5:6	24.2 (3-46)
Nieto (2021)	42	42	48 (23-71)	28:14	NR (6-156)^c^
Renteria (2023)^a^	35	38	50.1 (NR)	11:27	4 (NR)
Rodgers (2016)^a^	14	14	48 (37-61)	11:18	NR
Saliba (2014)	28	28	44 (27-60)	15:13	NR (2-NR)
Schwartz (2019)	21	21	47.9 (NR)	22:25	NR
Suresh (2024)	32	64	52.6^b^ (NR)	NR	9.4^b^ (NR)
Thomeer (2016)^a^	13	16	47.3 (28-61)	5:8	31.1 (3-95)
Tooker (2024)	57	63	48.0 (37-55)	29:28	25.9 (NR)
Totten (2022)	46	49	51.1 (NR)	27:19	NR
Tugrul (2022)	11	11	NR	NR	24 (18-42)
Wolfovitz (2019)	5	5	49.8 (21-66)^c^	NR	14.1 (7-46)^c^
Yang (2024)	410	410	49.7 (NR)	149:261	4.5 (NR)

Abbreviation: NR, not recorded.

a Studies that did not report symptoms, only audiometric outcomes or other clinical findings.

b Median.

c Characteristic not split by surgical approach.

### Audiological Outcomes

Presenting auditory symptoms and post-operative improvement of symptoms are reported in Table 2. The most commonly reported pre-operative symptoms were autophony (70.6%; n = 721/1021) and tinnitus (67.3%; n = 698/1037). Patient-reported audiologic improvement following SSCD repair was high [subjective hearing loss (53%; n = 200/378), aural fullness (62.6%; n = 324/517), autophony (76.5%; n = 552/721), and tinnitus (63.2%; n = 441/698)]. Audiometric PTA data are reported in Table 3.

**Table 2.** Pre-Operative Cochlear Symptoms and Post-Operative Improvement.

Study	Subjective hearing loss		Aural fullness		Autophony		Tinnitus	
	Pre- (n)	Improved post	Pre- (n)	Improved post	Pre- (n)	Improved post	Pre- (n)	Improved post
Trans-mastoidM								
Beyea (2012)	7	2					10	10
Bogle (2013)	12	7	9	3	6	1	10	3
deWolf (2021)	30	18	33	24	37	34	37	31
Eberhard (2024)					13	13	6	5
Gersdorff (2022)			19	18	29	28	18	11
Kontorinis (2021)			9	5	7	7	9	5
Ma (2015)	5	4	3	3	7	5	8	7
Nieto (2021)	6	3	5	4	7	7	6	5
Nogueira (2018)					16	16	14	14
Schwartz (2019)			17	11	40	38	24	19
Shaul (2024)	6	3	9	8	18	17	16	13
Tooker (2024)					28	26	25	20
Van Haesendonck (2016)					11	10	8	6
Wolfovitz (2019)	3	3	5	5	3	3	6	6
Zhao (2012)	1	1			9	9	2	1
All TM studies	79	46	109	81	231	214	199	156
Middle Cranial Fossa								
Baxter (2019)			25	22	32	30	28	24
Goddard (2014)			20	16	15	12	11	8
Lee (2020)	3	3	8	8	10	10	10	10
Nieto (2021)	36	27	25	24	20	17	34	29
Saliba 2014 (not split)			26	26	28	28	25	25
Schwartz (2019)			5	4	10	8	5	3
Suresh (2024)	16	9	22	19	20	12	15	6
Tooker (2024)					27	20	18	13
Totten (2022)^a^	20	17	27	24	34	2	31	28
Tugrul (2022)	11	11	11	11	11	11	11	11
Wolfovitz (2019)	2	2	1	1	1	1	3	3
Yang (2024)^a^	211	85	238	88	282	187	308	125
All MCF studies	299	154	408	243	490	338	499	285

Abbreviation: MCF, middle cranial fossa; TM, transmastoid.

a Numbers calculated from reported percentages with a total of 46 patients.

**Table 3.** Audiometric PTA for AC, BC, and ABG for All Reporting Studies.

Approach		Author	n (patients)	Pre-operative PTA		Post-operative PTA		Delta-PTA	
				Mean (dB)	SD (dB)	Mean (dB)	SD (dB)	Mean (dB)	SD (dB)
MCF	BC	Ellsperman (2021)	24, 19 (pre/post)	10.7	7.6	11.25	8.6	1.27	
		Goddard (2014)	16	15.6	14.1	16.2	17.1		
		Lin (2021)	43	7.4	6	5.6	6.6	4.9	11.3
		Nieto (2021)	42	17.86	21.89	18.7	21.6		
		Thomeer (2016)	16	10.8	7.8	10.9	6.8	−0.2	5.6
		Totten (2022; endoscopic)	9	13.5		19.8			
		Totten (2022; microscopic)	40	16.2		14.8			
	AC	Ellsperman (2021)	24, 19 (pre/post)	19.7	10.2	18.2	12.8	3.2	
		Goddard (2014)	23	21.1	21.6	22.5	16.7		
		Lin (2021)	43	20.4	12.6	23.5	18.5	3.1	13.4
		Nieto (2021)	42	32.7	30.9	29.3	25.6		
		Saliba (2014)	28	21.74		16.25			
		Schwartz (2019)	14	22.9				0	3.92
		Thomeer (2016)	16	21.7	10.1	17.2	7.1	4.5	5.3
		Totten (2022; endoscopic)	9	26		31.8			
		Totten (2022; microscopic)	40	26.5		22.7			
	ABG	Ellsperman (2021)	24, 19 (pre/post)	15.5	13.1	8.6	7.5		
		Goddard (2014)	14	9.3	7.1	7.9	7.6	1.7	
		Lin (2021)	43	13	9.6	17.9	16.9		
		Renteria (2023)	38	13.34	9.8	7.1	6.9		
		Thomeer (2016)	16	10.9	6.9	6.3	4	−4.7	6.9
		Totten (2022; endoscopic)	9	12.5		12			
		Totten (2022; microscopic)	40	10.4		7.8			
TM	BC	Creighton (2021)						−7.5	
		Ellsperman (2021)	26, 15 (pre/post)	17.9	16.5	17.4	12.9	−0.57	
		Lin (2021)	29	17.5	16	7.2	7	4.8	7
		Nieto (2021)	9	15	13.4	16.9	12.6		
		Shaul (2024)	23 (24 ears)	13.7	17	20.5	18		
		Van Haesendonck (2016)	12 (13 ears)	13.38	9.86	16.42	6.17	−2.75	7.36
		Zhao (2012)	11	14.32	20.53	19.5	21.33		
	AC	Creighton (2021)	6	4.3		16			
		Ellsperman (2021)	26, 15 (pre/post)	28.1	19.6	26.9	13.3	−1.4	
		Kontorinis (2021)	11	15.21	8.6	14.16	9.43	−1.04	4.32
		Lin (2021)	29	25.6	18.9	29.5	20	3.9	8.1
		Nieto (2021)	9	23.6	15.7	26.4	14		
		Schwartz (2019)	39					−0.79	5.39
		Shaul (2024)	23 (24 ears)	28.5		29.75		1.25	
		Van Haesendonck (2016)	12	24.83	13.86	21.17	9.77	3.58	6.68
		Zhao (2012)	11	35.45	25.3	35.72	27.95	0.27	5.9
	ABG	Ellsperman (2021)	26, 15 (pre/post)	22.8	11.1	18.7	9.6		
		Lin (2021)	29	8.1	6.4	22.3	18.4		
		Shaul (2024)	23 (24 ears)	11		6		−5	
		Van Haesendonck (2016)	12	11.41	10.45	5.00	5.38	6.17	7.76
		Zhao (2012)	11	23.82	16.26	20.5	14.92		

Abbreviation: ABG, air-bone gap; AC, air-conduction; BC, bone-conduction; MCF, middle cranial fossa; TM, transmastoid.

Meta-analysis using random-effect generalized linear models was completed and indicates a 60% to 95% likelihood of post-operative improvement of debilitating auditory symptoms following SSCD repair, regardless of the surgical approach utilized (Table 4). No statistically significant differences were observed in auditory symptom improvement when comparing the TM and MCF approaches (Table 4).

**Table 4.** Estimated Probability of Improvement/Resolution of Pre-Operative Symptoms Following SSCD Repair via the MCF and TM Approaches.

Symptom	MCF approach % (CI)	TM approach % (CI)	Adjusted odds ratio (CI)	P value
Subjective hearing loss	75.1% (55.7-87.9)	60.7% (40.4-77.9)	0.51 (0.16-1.64)	.26
Aural fullness	90.3% (77.8-96.1)	79.6% (58.7-91.4)	0.42 (0.11-1.64)	.21
Autophony	84.9% (63.7-94.7)	95.5% (86.2-98.6)	3.77 (0.74-19.15)	.11
Tinnitus	83.3% (69.3-91.7)	81.3% (67.9-89.9)	0.87 (0.31-2.49)	.79
Vertigo	81.8% (64.4-91.8)	78.9% (63.1-89.1)	0.83 (0.26-2.70)	.76
Non-vertiginous dizziness	67.8% (41.2-86.4)	81.6% (63.2-92.0)	2.10 (0.51-8.61)	.30
Oscillopsia	61.7% (44.8-76.1)	91.1% (75.1-97.2)	6.40 (1.66-24.66)	.007*
Tullio phenomenon	87.3% (72.8-94.7)	89.1% (77.6-95.1)	1.19 (0.35-4.10)	.77
Hennebert sign	83.1% (45.0-96.7)	82.8% (63.7-93.0)	0.98 (0.12-7.68)	.98

Adjusted odds of symptom resolution for repair using the TM approach compared to the MCF approach based on random-effect model analysis noted.

Abbreviations: MCF, middle cranial fossa; SSCD, superior semicircular canal dehiscence; TM, transmastoid.

* *P* < .05.

**Figure 2.**
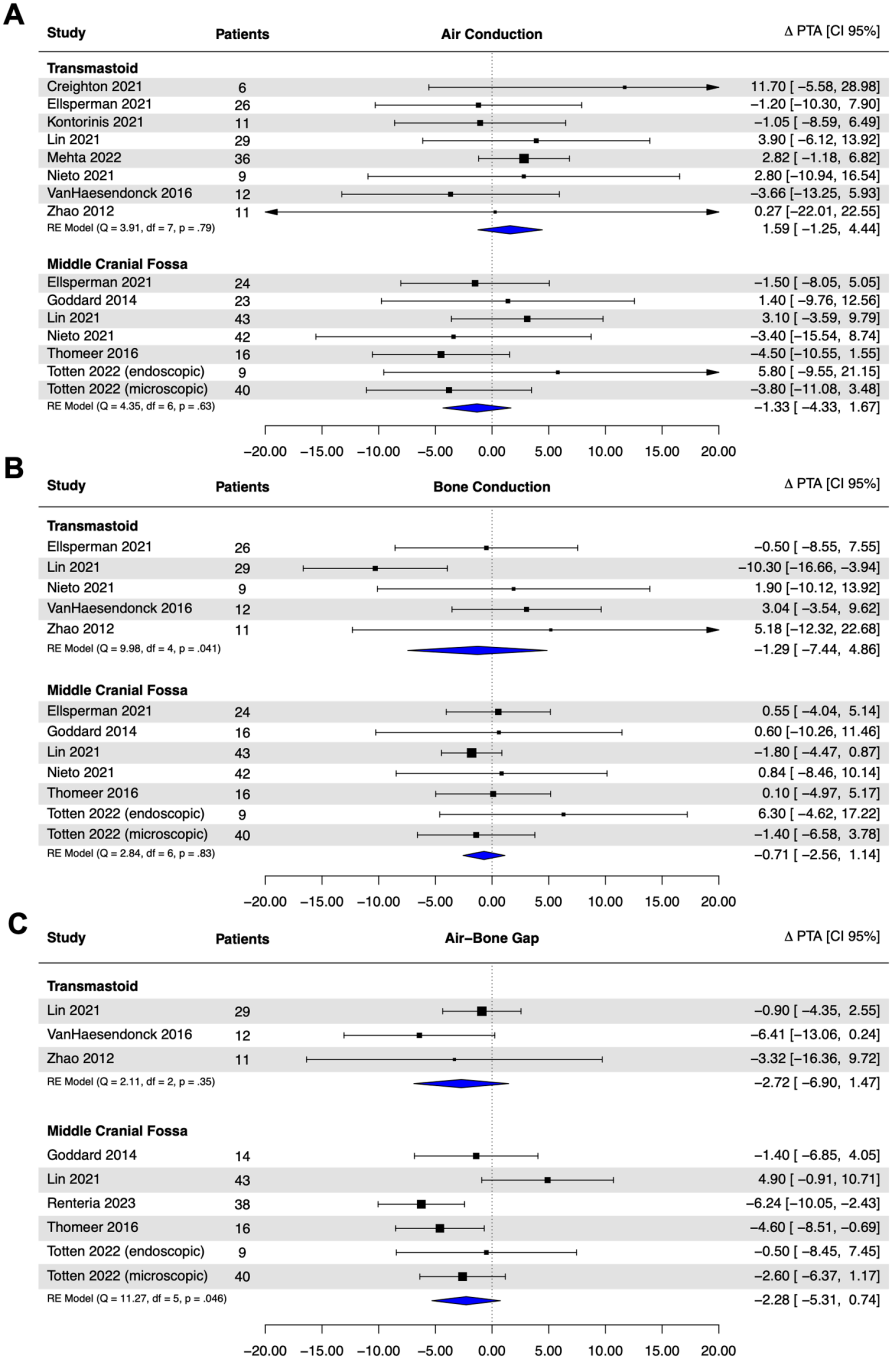
Post-operative threshold changes for pure-tone averages to (A) air-conduction audiometry. (B) Bone-conduction audiometry. (C) ABG. Results of random-effect models presented with pooled estimates of threshold changes. Negative values indicate improvement in audiometric thresholds and a decrease in ABG. ABG, air-bone gap.

Meta-analyses of AC PTA, BC PTA, and ABG PTA were completed. In total, 16 studies were included in the meta-analysis, yielding audiometric data on 403 patients (TM, n = 128; MCF, n = 275). Regardless of the surgical approach, no significant post-operative threshold changes in AC PTA, BC PTA, or ABG PTA were noted (Figure 2). No significant differences in post-operative changes in PTA thresholds were found between surgical approaches for AC thresholds [ΔPTA_TM_ − ΔPTA_MCF_ = +2.92 dB; 95% CI = (−1.21 to 7.05); *P* = .17], BC thresholds [ΔPTA_TM_ − ΔPTA_MCF_ = −2.23 dB; 95% CI = (−6.55 to 2.07); *P* = .31], or ABG [ΔPTA_TM_ − ΔPTA_MCF_ = −2.20; 95% CI = (−6.86 to 2.46 dB); *P* = .36].

### Vestibular Testing Outcomes

Vestibular symptoms were less frequently reported within studies, with many studies only reporting on the presence and absence of the Tullio phenomenon and/or non-vertiginous dizziness (Table 5). When reported, symptomatic improvement following SSCD repair was high across all vestibular symptoms: vertigo (70.9%; n = 220/310), non-vertiginous dizziness (54.8%; n = 305/556), oscillopsia (63.7%; n = 125/196), Tullio phenomenon (84.3%; n = 189/224), and the Hennebert sign (82.5%; 52/63). cVEMP were infrequently reported in the literature. Nine studies reported cVEMP thresholds on a total of 199 patients (TM = 67, MCF = 132; Table 6). Descriptively, cVEMP impairment largely appeared to resolve following surgical correction. The DHI score was also infrequently reported, with only 6 studies reporting DHI averages. Pre-operative DHI scores (mean = 45.3; range = 38.8-56.7) fell largely fell within the moderate to severe range,^44^ with improvement of burden of dizziness symptoms post-operatively, consistent with the high rates of symptomatic improvement in dizziness and vertigo (Table 7).

**Table 5.** Vestibular Symptoms and Post-Operative Outcomes.

Study	Vertigo		Non-vertiginous dizziness		Oscillopsia		Tullio phenomenon		Hennebert sign	
	Pre- (n)	Improved post	Pre- (n)	Improved post	Pre- (n)	Improved post	Pre- (n)	Improved post	Pre- (n)	Improved post
TM										
Beyea (2012)										
Bogle (2013)	3	1	20	8			5	3	3	2
deWolf (2021)			26	16			30	26	23	20
Eberhard (2024)							11	11	11	11
Ellsperman (2022)										
Gersdorff (2022)	24	19	24	19	11	9	19	17		
Kontorinis (2021)					11	11				
Lundy (2011)			37	34						
Ma (2015)	6	5	5	5	1	1	6	4	6	5
Nieto (2021)			9	8	6	5				
Nogueira (2018)			18	18						
Schwartz (2019)	29	18					19	17		
Shaul (2024)			18	10	4	4	11	11		
Tooker (2024)	49	43								
Van Haesendonck (2016)							5	4	5	2
Wolfovitz (2019)	3	3	6	6						
Zhao (2012)	2	2					3	3		
All TM studies	97	71	158	119	33	30	109	96	48	40
MCF										
Baxter (2019)			13	9			27^a^	27^a^	27^a^	27^a^
Goddard (2014)							8	8	11	8
Lee (2020)			8	8			4^a^	4^a^	4^a^	4^a^
Nieto (2021)			37	29	16	13	17	14		
Schwartz (2019)	6	4					10	8		
Suresh (2024)			20	9	6	3	18	15		
Tooker (2024)	49	43								
Totten (2022)^a^					14	10	29	17		
Tugrul (2022)	11	11								
Wolfovitz (2019)	3	3								
Yang (2024)	141	88	320	131	127	69				
All MCF studies	210	149	398	186	163	95	113	93	15	12

Abbreviation: MCF, middle cranial fossa; TM, transmastoid.

a Study reported together.

**Table 6.** cVEMP Results Pre- and Post-Operatively for All Reporting Studies.

Study	N patients	Pre-operative cVEMP	Post-operative cVEMP
Transmastoid			
Bogle (2013)	20	152.99^a^	NR
Gersdorff (2022)	11	100.45	121.36
Nogueira (2018)	21	59.1	87.3
Rodgers (2016)	15	60.1	89.1
Middle cranial fossa			
Lee (2021)	11	55.7	77.6
Minor (2005)	30	81	NR
Renteria (2023)	35	64.2	81.7
Rodgers (2016)	14	69.3	87.3
Saliba (2014)	28	61	80
Thomeer (2016)	14	76.1	94.4

Abbreviation: cVEMP, cervical vestibular evoked myogenic potential.

a cVEMP thresholds measured in microvolts.

**Table 7.** DHI Results Pre- and Post-Operatively for All Reporting Studies.

Study	N patients	Pre-operative DHI		Post-operative DHI	
		Mean (SD)	Range	Mean (SD)	Range
Transmastoid					
Allsopp (2019)	10	38.8	2-88	16	2-38
Bogle (2013)	20	48^a^ (IQR: 28-56)		33^a^ (IQR: 19-50)	
Dewolf (2021)	37	45.9 (4.4)		27.4 (3.05)	
Ellsperman (2022)	28	39.4	10-86	44.7	34-86
Kontorinis (2021)	11	56.7	22-84	25.83	10-46
Middle cranial fossa					
Ellsperman (2022)	24	45.7	12-86	32.4	8-76

Abbreviation: DHI, Dizziness Handicap Inventory.

a Median.

Meta-analysis on post-surgical vestibular symptom improvement was completed and indicates that patients can expect a 61.7% to 91.1% likelihood of post-operative improvement of debilitating vestibular symptoms following SSCD repair (Table 4). As shown in Table 4, post-operative symptomatic improvement rates did not significantly differ between surgical approaches for vertigo, non-vertiginous dizziness, Tullio phenomenon, or Hennebert sign. Only improvement rates of oscillopsia were noted between surgical approaches, with patients undergoing surgery with a TM approach being more likely to report improvement compared to the MCF approach [91.1% vs 61.7%; adjusted OR [aOR] = 6.40; 95% CI = (1.66-24.66); *P* = .007].

## Discussion

This systematic review and meta-analysis show surgical intervention in SSCDS results in high rates of post-operative symptom improvement, regardless of whether a TM or MCF approach is utilized. Only post-operative symptom improvement of oscillopsia was found to be different between approaches, with the TM patients being more likely than the MCF cohort to report improvement. Analysis of audiometric data indicates no significant mean threshold shift from pre-operative baseline in AC or BC PTAs. No significant differences were observed in post-operative AC PTA, BC PTA, or ABG PTAs between surgical approaches. Taken together, these results indicate minimal differences in post-operative auditory and vestibular outcomes in SSCDS correction between TM and MCF approaches.

It is very important to establish if a surgical approach to SCDS is superior in order to direct best practice. While similar outcomes between the TM and MCF approach is not unexpected, it was important to determine overtly.

In addition, while post-operative complications are generally transient, reporting is sporadic. In context, this suggests no differences in overall outcomes between surgical approaches, with both being effective. Given that the MCF requires a full craniotomy to gain access to the dehiscence, the MCF approach with full craniotomy is associated with longer hospital admissions and lengthened duration of recovery. The TM approach is often done in an outpatient setting.^23,45,46^ In contrast, TM approach may not permit direct visualization of the dehiscence, which based on the data does not appear to change operative outcomes. Minimally invasive MCF approaches such as the “keyhole” craniotomy utilize a smaller incision and are expected to require hospital admissions of similar duration to TM approaches, although direct comparison was not completed within the current study.

Previous systematic reviews have found no significant differences in outcomes based on whether the dehiscence is plugged, resurfaced, or capped.^6,45^ The impact of the method of dehiscence closure, as well as the material used in the closure, was not collected in the current review, due in part to heterogeneity in reporting. It is possible that the materials used for dehiscence closure, or whether the dehiscence is plugged or capped, had a significant impact on overall outcomes and skewed the comparison between surgical approaches.

There are 2 possible explanations for the statistically significant improvement in post-operative oscillopsia when utilizing the TM approach. The first is the sample size. Overall, the sample size was quite limited, with 163 MCF patients and 33 TM patients; only one other symptom had a smaller sample size. In addition, almost 80% of all MCF patients originated from a single study.^34^ A second possible explanation is that this is a false positive, given the low sample size and the presence of multiple comparisons completed within our study. While there is an argument as to whether to correct for multiple comparisons, the approach used in this study was determined a priori to not do so to liberally permit identification of potential differences observed between surgical approaches.^47^ However, the authors are unable to provide a physiological explanation for differing post-operative oscillopsia improvement rates while all other vertiginous symptoms were non-different between approaches.

Several studies included in the systematic review had audiometric outcomes available, however, they were either frequency specific, only available within figures, or the PTA was otherwise unable to be calculated.^19,21,26,31,33-35,37,39,41^ Of the studies included within the meta-analysis, post-operative average AC and BC thresholds did not significantly differ from pre-operative values following surgery, regardless of surgical approach. Marginal ABG closure was observed following surgery utilizing the TM approach, and no differences were observed between surgical approaches. This is in direct contrast to the *subjective* improvements in hearing loss reported by 60% and 74% of patients following surgery using the TM and MCF approach, respectively. Although surgical correction is expected to close the ABG by decreasing BC hyperacusis, several previous studies have reported a lack of significant improvement.^23,48,49^ This does not, however, indicate a lack of benefit. Yang et al found that although a lack of an ABG pre-operatively was associated with decreased symptom resolution, a lack of ABG closure was not.^48^ It is possible that the apparent discordance between subjective hearing loss improvement and audiometric thresholds may be due to an improvement in the perceived quality of sound, rather than the thresholds themselves. It is also possible that PTA was not an adequately sensitive measure to implicate ABG closure, as the largest changes are expected within the low frequencies. Finally, it is possible that the threshold shifts are not permanent or may change over time, which has been reported previously, and may be why some groups do not suggest surgical intervention solely for audiometric outcomes.^45,50^

In the absence of differences in patient subjective and objective measures, one could argue for consideration of the TM approach owing to less brain retraction and shorter hospitalization. It is important to specifically identify that there were no differences in reported complications between approaches, yet this was not well documented in many studies. Medical care is a limited resource and hospitalization has real economic implications for patients and medical systems.

This review has its limitations. The first of which is the considerable heterogeneity in outcomes reported and the way that they are reported. This heterogeneity significantly decreased the number of patients that were able to be included accurately within the meta-analysis, which may bias our findings. This heterogeneity in outcomes and follow-up duration, which has been noted previously,^51^ decreases the validity and generalizability of secondary analysis. To remedy this, the authors recommend standardized reporting of a battery of symptoms and complications—regardless of if the patients report them or not—as well as robust audiometric data reporting.

Another very important limitation is that the majority of patients within the review were non-randomized. At least 1 study explicitly stated that they chose a surgical approach prior to surgery based on patient characteristics, which may skew results. Further, as already identified, a large contribution of the MCF data originated from a single institution. However, analysis was re-run without the patients reported by Yang et al did not meaningfully change. Finally, another important limitation is that while the surgical approach employed was the focus of this work, there was no consideration of how the SCD was managed. There are several broad categories of repair, including resurfacing and plugging. This differentiation is likely important; however, subgroup analysis was not performed.

## Conclusion

Surgical intervention for debilitating SSCDS symptoms has excellent rates of post-operative auditory and vestibular symptom improvement with minimal differences between TM and MCF approaches. Average post-operative audiometric PTA thresholds were similar to pre-operative levels. The choice of surgical approach should be decided based on the surgeon's preference and individual patient factors.

## Supplemental Material

sj-docx-1-ohn-10.1177_19160216261435611 – Supplemental material for Comparison of Outcomes in Superior Canal Dehiscence Surgery Using Either Transmastoid or Middle Fossa Surgical Approaches: A Systematic Review and Meta-Analysis Supplemental material, sj-docx-1-ohn-10.1177_19160216261435611 for Comparison of Outcomes in Superior Canal Dehiscence Surgery Using Either Transmastoid or Middle Fossa Surgical Approaches: A Systematic Review and Meta-Analysis by Matthew Urichuk, Jason Azzi, Ben Woldu, Armon Hadian and Jordan Hochman in Journal of Otolaryngology - Head & Neck Surgery

sj-docx-2-ohn-10.1177_19160216261435611 – Supplemental material for Comparison of Outcomes in Superior Canal Dehiscence Surgery Using Either Transmastoid or Middle Fossa Surgical Approaches: A Systematic Review and Meta-Analysis Supplemental material, sj-docx-2-ohn-10.1177_19160216261435611 for Comparison of Outcomes in Superior Canal Dehiscence Surgery Using Either Transmastoid or Middle Fossa Surgical Approaches: A Systematic Review and Meta-Analysis by Matthew Urichuk, Jason Azzi, Ben Woldu, Armon Hadian and Jordan Hochman in Journal of Otolaryngology - Head & Neck Surgery
