# Supplementary material for: Comparison of Outcomes in Superior Canal Dehiscence Surgery Using Either Transmastoid or Middle Fossa Surgical Approaches: A Systematic Review and Meta-Analysis
Source: J Otolaryngol Head Neck Surg. 2026 Mar 30;55:19160216261435611. doi: 10.1177/19160216261435611 (PMC13036347; doi:10.1177/19160216261435611)
Supplement: sj-docx-1-ohn-10.1177_19160216261435611 – Supplemental material for Comparison of Outcomes in Superior Canal Dehiscence Surgery Using Either Transmastoid or Middle Fossa Surgical Approaches: A Systematic Review and Meta-Analysis [file sj-docx-1-ohn-10.1177_19160216261435611.docx]

**Supplemental Material 1: Search String**

**Medline**

"Superior semicircular canal dehiscence syndrome" OR"Semicircular canal dehiscence syndrome" OR "Superior semi-circular canal dehiscence syndrome" OR "Superior semicircular duct dehiscence" OR "Semicircular duct dehiscence" OR "Semicircular dehiscence syndrome" OR "Semi-circular duct dehiscence" OR "Semi-circular dehiscence syndrome" OR "Semicircular Canal Dehiscence" OR "Semi-circular Canal Dehiscence" OR "SSCD" OR "SCDS" OR Semicircular Canal Dehiscence/

AND

(

("middle fossa approach" OR "middle fossa craniotomy" OR "middle cranial fossa approach" OR "middle cranial fossa craniotomy" OR "middle fossa" OR "middle cranial fossa" OR "transmastoid approach" OR "trans-mastoid approach" OR "transmastoid" OR "trans-mastoid" OR "plug" OR "plugging" OR "recap" OR "recapping" OR "resurface" OR "resurfacing" OR "microscopic" OR "endoscopic")

OR

(Surgical Procedures, Operative/ OR Craniotomy/ OR Endoscopy/ OR Microscopy/)

OR

("Surgical Complications" OR "Complications" OR "postoperative complications" OR "post-operative complications" OR "intracranial complications") OR (Postoperative complications/)

OR

"Tinnitus" OR "pulsatile tinnitus" OR "Hyperacusis" OR "autophony" OR "phonophobia" OR "hearing loss" OR "hearing impairment" OR "hearing" OR "hearing disorder" OR "hearing function" OR "aural fullness" OR "pressure" OR "vertigo" OR "dizziness" OR "imbalance" OR

"oscillopsia" OR "Tullio phenomenon" OR "sound-induced dizziness" OR "sound-induced vertigo"

OR

Tinnitus/ OR Hyperacusis/ OR Hearing loss/ OR Vertigo/ OR Pressure/

)

**EMBASE:**

"Superior semicircular canal dehiscence syndrome" OR"Semicircular canal dehiscence syndrome" OR "Superior semi-circular canal dehiscence syndrome" OR "Superior semicircular duct dehiscence" OR "Semicircular duct dehiscence" OR "Semicircular dehiscence syndrome" OR "Semi-circular duct dehiscence" OR "Semi-circular dehiscence syndrome" OR "Semicircular Canal Dehiscence" OR "Semi-circular Canal Dehiscence" OR "SSCD" OR "SCDS"

OR semicircular canal dehiscence/

AND

(

("middle fossa approach" OR "middle fossa craniotomy" OR "middle cranial fossa approach" OR "middle cranial fossa craniotomy" OR "middle fossa" OR "middle cranial fossa" OR "transmastoid approach" OR "trans-mastoid approach" OR "transmastoid" OR "trans-mastoid" OR "plug" OR "plugging" OR "recap" OR "recapping" OR "resurface" OR "resurfacing" OR "microscopic" OR "endoscopic")

OR

Craniotomy/ OR middle cranial fossa/ OR surgical approach/ OR temporal bone/

OR

("Surgical Complications" OR "Complications" OR "postoperative complications" OR "post-operative complications" OR "intracranial complications")

OR

postoperative complication/

OR

"Tinnitus" OR "pulsatile tinnitus" OR "Hyperacusis" OR "autophony" OR "phonophobia" OR "hearing loss" OR "hearing impairment" OR "hearing" OR "hearing disorder" OR "hearing function" OR "aural fullness" OR "pressure" OR "vertigo" OR "dizziness" OR "imbalance" OR

"oscillopsia" OR "Tullio phenomenon" OR "sound-induced dizziness" OR "sound-induced vertigo"

OR

Nystagmus/ OR Vertigo/ OR Dizziness/ OR vestibular disorder/ OR vestibular system/ OR positional dizziness/ OR hyperacusis/ OR tinnitus/ OR hearing disorder/ OR hearing impairment/ OR hearing loss/ OR phonophobia/ OR oscillopsia/

**SCOPUS + Web of Science:**

"Superior semicircular canal dehiscence syndrome" OR"Semicircular canal dehiscence syndrome" OR "Superior semi-circular canal dehiscence syndrome" OR "Superior semicircular duct dehiscence" OR "Semicircular duct dehiscence" OR "Semicircular dehiscence syndrome" OR "Semi-circular duct dehiscence" OR "Semi-circular dehiscence syndrome" OR "Semicircular Canal Dehiscence" OR "Semi-circular Canal Dehiscence" OR "SSCD" OR "SCDS"

AND

("middle fossa approach" OR "middle fossa craniotomy" OR "middle cranial fossa approach" OR "middle cranial fossa craniotomy" OR "middle fossa" OR "middle cranial fossa" OR "transmastoid approach" OR "trans-mastoid approach" OR "transmastoid" OR "trans-mastoid" OR "plug" OR "plugging" OR "recap" OR "recapping" OR "resurface" OR "resurfacing" OR "microscopic" OR "endoscopic")

OR

("Surgical Complications" OR "Complications" OR "postoperative complications" OR "post-operative complications" OR "intracranial complications")

OR

"Tinnitus" OR "pulsatile tinnitus" OR "Hyperacusis" OR "autophony" OR "phonophobia" OR "hearing loss" OR "hearing impairment" OR "hearing" OR "hearing disorder" OR "hearing function" OR "aural fullness" OR "pressure" OR "vertigo" OR "dizziness" OR "imbalance" OR

"oscillopsia" OR "Tullio phenomenon" OR "sound-induced dizziness" OR "sound-induced vertigo"

)

**CINAHL:**

"Superior semicircular canal dehiscence syndrome" OR"Semicircular canal dehiscence syndrome" OR "Superior semi-circular canal dehiscence syndrome" OR "Superior semicircular duct dehiscence" OR "Semicircular duct dehiscence" OR "Semicircular dehiscence syndrome" OR "Semi-circular duct dehiscence" OR "Semi-circular dehiscence syndrome" OR "Semicircular Canal Dehiscence" OR "Semi-circular Canal Dehiscence" OR "SSCD" OR "SCDS" OR

(MH "Semicircular Canal Dehiscence+")

AND

(

("middle fossa approach" OR "middle fossa craniotomy" OR "middle cranial fossa approach" OR "middle cranial fossa craniotomy" OR "middle fossa" OR "middle cranial fossa" OR "transmastoid approach" OR "trans-mastoid approach" OR "transmastoid" OR "trans-mastoid" OR "plug" OR "plugging" OR "recap" OR "recapping" OR "resurface" OR "resurfacing" OR "microscopic" OR "endoscopic")

OR

(MH "Surgical Procedures, Operative+") OR (MH "Craniotomy+") OR (MH "Endoscopy+") OR (MH "Microscopy+")

OR

("Surgical Complications" OR "Complications" OR "postoperative complications" OR "post-operative complications" OR "intracranial complications")

OR

(MH "Postoperative complications+")

OR

"Tinnitus" OR "pulsatile tinnitus" OR "Hyperacusis" OR "autophony" OR "phonophobia" OR "hearing loss" OR "hearing impairment" OR "hearing" OR "hearing disorder" OR "hearing function" OR "aural fullness" OR "pressure" OR "vertigo" OR "dizziness" OR "imbalance" OR

"oscillopsia" OR "Tullio phenomenon" OR "sound-induced dizziness" OR "sound-induced vertigo"

OR

(MH "Tinnitus+") OR (MH "Hyperacusis+") OR (MH "Hearing loss+") OR (MH "Vertigo+") OR (MH "Pressure+")

)
