# Supplementary material for: Comparison of Outcomes in Superior Canal Dehiscence Surgery Using Either Transmastoid or Middle Fossa Surgical Approaches: A Systematic Review and Meta-Analysis
Source: J Otolaryngol Head Neck Surg. 2026 Mar 30;55:19160216261435611. doi: 10.1177/19160216261435611 (PMC13036347; doi:10.1177/19160216261435611)
Supplement: sj-docx-2-ohn-10.1177_19160216261435611 – Supplemental material for Comparison of Outcomes in Superior Canal Dehiscence Surgery Using Either Transmastoid or Middle Fossa Surgical Approaches: A Systematic Review and Meta-Analysis [file sj-docx-2-ohn-10.1177_19160216261435611.docx]

| Author | Selection | | | | Comparability | Outcome | | | Total |
| --- | --- | --- | --- | --- | --- | --- | --- | --- | --- |
|  | Representation of exposed cohort | Representation of non-exposed cohort | Ascertainment of exposure | Absence of outcome prior to exposure | design and analysis | Assessment of outcome | Duration of follow-up | Adequacy of follow-up |  |
| AlAfif 2019 | 1 | 0 | 1 | 1 | 0 | 1 | 1 | 1 | 6 |
| Baxter 2019 | 1 | 0 | 1 | 1 | 0 | 1 | 1 | 1 | 6 |
| Beyea 2012 | 1 | 0 | 1 | 1 | 0 | 1 | 1 | 1 | 6 |
| Bogle 2013 | 1 | 0 | 1 | 1 | 0 | 1 | 1 | 1 | 6 |
| deWolf et al. 2021 | 1 | 0 | 1 | 1 | 0 | 1 | 1 | 1 | 6 |
| Eberhard et al. 2024 | 1 | 0 | 1 | 1 | 0 | 1 | 1 | 1 | 6 |
| Ellsperman et al. 2022 | 1 | 0 | 1 | 1 | 0 | 1 | 1 | 1 | 6 |
| Gersdorff et al. 2022 | 1 | 0 | 1 | 1 | 0 | 1 | 1 | 1 | 6 |
| Goddard 2014 | 1 | 0 | 1 | 1 | 0 | 1 | 1 | 1 | 6 |
| Kontorinis et al. 2021 | 1 | 0 | 1 | 1 | 0 | 1 | 1 | 1 | 6 |
| Lee 2020 | 1 | 0 | 1 | 1 | 0 | 1 | 1 | 1 | 6 |
| Lundy 2011 | 1 | 0 | 1 | 1 | 0 | 1 | 1 | 1 | 6 |
| Ma 2015 | 1 | 0 | 1 | 1 | 0 | 1 | 1 | 1 | 6 |
| Nieto et al. 2021 | 1 | 0 | 1 | 1 | 0 | 1 | 1 | 1 | 6 |
| Nogueira 2018 | 1 | 0 | 1 | 1 | 0 | 1 | 1 | 1 | 6 |
| Saliba 2014 | 1 | 0 | 1 | 1 | 0 | 1 | 1 | 1 | 6 |
| Schwartz 2019 | 1 | 0 | 1 | 1 | 0 | 1 | 1 | 1 | 6 |
| Shaul et al. 2024 | 1 | 0 | 1 | 1 | 0 | 1 | 1 | 1 | 6 |
| Suresh et al. 2024 | 1 | 0 | 1 | 1 | 0 | 1 | 1 | 1 | 6 |
| Tooker et al. 2024 | 1 | 0 | 1 | 1 | 0 | 1 | 1 | 1 | 6 |
| Totten et al. 2022 | 1 | 0 | 1 | 1 | 0 | 1 | 1 | 1 | 6 |
| Tugrul et al. 2022 | 1 | 0 | 1 | 1 | 0 | 1 | 1 | 1 | 6 |
| Van Haesendonck 2016 | 1 | 0 | 1 | 1 | 0 | 1 | 1 | 1 | 6 |
| Wolfovitz 2019 | 1 | 0 | 1 | 1 | 0 | 1 | 1 | 1 | 6 |
| Yang 2024 | 1 | 0 | 1 | 1 | 0 | 1 | 1 | 1 | 6 |
| Zhao 2012 | 1 | 0 | 1 | 1 | 0 | 1 | 1 | 1 | 6 |

**Supplemental Material 2:** Newcastle-Ottawa Scale quality score for assessing the quality of nonrandomized studies
